# Setting Research Priorities for HIV/AIDS-related research in a post-graduate training programme: lessons learnt from the Nigeria Field Epidemiology and Laboratory Training Programme scientific workshop

**DOI:** 10.11604/pamj.2014.18.262.4804

**Published:** 2014-07-30

**Authors:** Gabriele Poggensee, Ndadilnasiya Endie Waziri, Adebobola Bashorun, Patrick Mboya Nguku, Olufunmilayo Ibitola Fawole, Kabir Sabitu

**Affiliations:** 1 Nigeria Field Epidemiology and Laboratory Training Programme, Abuja, Nigeria; 2 Epidemiology and Medical Statistics, Faculty of Public Health, College of Medicine, University of Ibadan, Nigeria; 3 Department of Community Medicine, Ahmadu Bello University, Zaria, Nigeria; 4 HIV/AIDS Division, Federal Ministry of Health, Abuja, Nigeria

**Keywords:** Research priorities, HIV/AIDS, training programme, field epidemiology, workshop, Nigeria

## Abstract

In Nigeria the current prevalence of HIV is 4.1% with over 3.5 million infected and estimated 1.5 million in need of anti-retroviral treatment. Epidemiological and implementation studies are necessary for monitoring and evaluation of interventions. To define research areas which can be addressed by participants of the Nigeria Field Epidemiology and Training Programme (NFELTP) a workshop was held in April 2013 in Abuja, Nigeria. Priority research areas were identified using criteria lists for ranking of the relevance of research questions. Based on a research matrix, NFELTP residents developed the aims and objectives, study design for HIV-related research proposals. This workshop was the first workshop held by the NFELTP to establish an inventory of research questions which can be addressed by the residents within their training period. This inventory will help to increase HIV/AIDS-related activities of NFELTP which are in accordance with research needs in Nigeria and PEPFAR objectives.

## Introduction

Although HIV prevalence is much lower in Nigeria than in other African countries such as South Africa and Zambia, the size of Nigeria's population (estimated population 162 million) means that by the end of 2009, there were an estimated 3.3 million people living with HIV [1]. The trend of HIV prevalence among women attending ante-natal has been dynamic with a steady increase from 1.8% (1991) to 4.6% (2007). The current prevalence is 4.1% (2012) with over 3.5 million infected and estimated 1.5 million are in need of anti-retroviral treatment. The epidemic is generalized, however, showing wide geographical variations. In response to the epidemic HIV prevention, care and treatment have scaled up in the last decade; however, the barriers to effectively replicate evidence-based interventions need to be investigated. The National Strategic Framework aims amongst others to conduct research in order to ensure evidence-based interventions [1]. Implementation research, epidemiological studies and impact studies are said to be necessary for monitoring and evaluation of interventions [2].

The Field Epidemiology and Laboratory Training Program (FELTP) is a two-year master's level training in applied /field epidemiology. FELTP serves to build local capacity in order to improve and strengthen countries’ public health systems and infrastructure [3]. In Nigeria, the Field Epidemiology Laboratory Training Program (NFELTP) has been working to build capacity in the public health workforce since 2008.

With support from the Federal Ministry of Health (FMOH), US Presidents Emergency Plans for Aids Relief (PEPFAR) {through the Centers for Disease Control and Prevention (CDC)} and two indigenous leading Nigerian universities, the NFELTP trains field public health laboratory, medical epidemiology, and veterinary epidemiology residents to work in leadership and technical positions in the Federal Ministries of Health (FMOH) and Agriculture and Rural Development (FMARD) in addition to leadership and technical positions at the state level. This two-year program helps improve public health systems within the country by increasing knowledge and skills in field epidemiology and laboratory epidemiology and management by building a cadre of skilled and well-trained health professionals (www.nigeria-feltp.net).

NFELTP has assisted in the detection, investigation and response of public health emergencies including disease outbreaks. Surveillance system evaluations and secondary data analyses have been carried out by NFELTP residents. HIV/AIDS-related research activities cover the evaluation of HIV surveillance systems in different states, HIV epidemiology (risk factors for HIV in different zones and high risk groups), prevention (acceptance of prevention of mother-to-child transmission of HIV (PMTCT) services), and HIV care (opportunistic infections).

However, there is a need to develop a comprehensive inventory of priority research areas of interest. The inventory will integrate the research needs identified by stakeholders involved in HIV prevention and care in Nigeria. Furthermore, the inventory will help for future NFELTP residents to decide on research topics of public health relevance in the early phase of their training. Duplications of research not adding new knowledge will thus be omitted.

In order to define research areas and research questions which can be worked on within the frame of the programme a workshop was held from 15th – 17th April 2013 in Abuja, Nigeria. Here we report the results of the workshop. The workshop aimed to encourage and support NFELTP residents to develop HIV/AIDS-related relevant research topics.

The expected outcomes were: 1) development of an inventory of relevant HIV/AIDS-related research topics which can be addressed by NFELTP residents; 2) development of draft study proposals by (future) residents.

Altogether 54 participants attended the workshop. From the Nigeria Field Epidemiology and Laboratory Training Program 44 residents, graduates, academic and program supervisor participated having clinical, laboratory, epidemiological and program working experience in HIV/AIDS. Furthermore, external experts from the National AIDS and STI Control Programme, Nigeria Centre for Disease Control, Federal Ministry of Health; AIDS Prevention Initiative in Nigeria (APIN); Center of Disease Control and Prevention Nigeria and Aminu Kano Teaching Hospital university, Kano, were invited as keynote speakers on treatment, care and support, HIV monitoring and evaluation, prevention of mother-to-child transmission (PMTCT) of HIV and Early Infant Diagnosis of HIV (EID), HIV epidemiology and surveillance and HIV/AIDS operational research. The first day of the workshop consisted of plenary sessions where the research setting was defined for each area. This was followed by deliberations in subgroups of participants with special interest in the specific topic area. The groups were moderated by the external experts and NFELTP academic and programme supervisors. The views of the participants and experts with regard to HIV/AIDS-related research gaps and research needs in Nigeria were assessed in a systematic manner using the following methods.

Prior to the workshop date, desk review of available literatures was conducted to better understand the priority research and gaps. Using a semi-structured questionnaire the workshop participants were also asked before the workshop to rate the relevance of HIV/AIDS related research questions. The questionnaire consisted of two parts: i) rating of broad research areas: prevention, HIV care, PMTCT, support, capacity building, monitoring & evaluation, data quality assurance and epidemiology; ii) rating of specific research questions on HIV prevention, PMTCT, epidemiology, HIV and tuberculosis, HIV care, HIV support, monitoring, evaluation and data usage. The development of the questionnaire was based on the outputs of literature search using PubMed and google scholar with the following keyword: research priorities, HIV/AIDS, Africa, resource-limited setting. The items were rated using the following scale: strong need (1), need (2), less need (3), no need (4) and undecided (0). For each item an average was calculated and the percentages of undecided answers were given. Additionally workshop participants could include in each area suggestions of relevant research questions. At the end of the first workshop day an inventory of research questions following the broad topic outline of the questionnaire was established based on the inputs from participants by questionnaire and discussion.

A criteria list for the ranking of the relevance of HIV-related research questions was developed including criteria on answerability and ethics, efficacy and impact, deliverability, affordability, scalability and sustainability, health systems, partnership and community involvement, equity achieved by research outputs. Points between 0 (low score) and 10 (high score) could be given to 15 questions. For each research question an average score was calculated.

Residents were asked to submit concept notes for an HIV/AIDS-related research proposal. Based on a research matrix, residents discussed in the working groups the aims and objectives, the study design and data sources for proposed research ideas. The outputs for individual research topics were presented and discussed in the plenum.

## Outcomes of the workshop

### Ranking of HIV/AIDS-relating research questions

The pre-workshop questionnaire was used to assess the ranking of research areas and specific research questions. Altogether 29 questionnaires were returned (return rate: 53%). The 20 highest ranking of research areas are summarized in Table 1.

**Table 1:** Summary of the 20 HIV/AIDS-research questions with the highest average scores based on the pre-workshop questionnaire (lines highlighted in grey: more than 10% of the respondents were undecided to give high or low priority).

Research areas	Topics	Undecided (%)	Average
General	Implementation research	6,9	1,07
General	HIV prevention, general	0,0	1,24
HIV andtuberculosis	Studies on provision of screening of TB patients for HIV	0,0	1,21
HIV andtuberculosis	Identification of best strategies for the co-management of TB and HIV care	0,0	1,29
HIV Care	Paediatriccoverage	3,7	1,14
HIV Care	Identification of socio-economic, behavioural, cultural, structural and other factors that have an impact on ART access and can be influenced by the programme	3,7	1,17
HIV epidemiology	Proportion of individuals becoming newly infected (incidence)	3,6	1,11
HIV prevention	Evaluation of combination of prevention approaches	3,4	1,24
Monitoring, datausage	Comprehensive inventory of publicly available data on HIV/AIDS	7,1	1,24
Monitoring, datausage	Studies on utilization of data	0,0	1,28
PMTCT	Evaluation of combination of prevention approaches	0,0	1,28
PMTCT	Early infant diagnosis – what is the proportion of mothers receiving test results	7.1	1.32
HIV care	Screening for cervical intraepithelial lesions (PAP)	25,0	1,14
HIV care	Contributionof private sector	14,8	1,28
HIV epidemiology	Numbers of individuals requiring ART	10,7	1,29
HIV support	Have workplace programs affected stigma and discrimination at the workplace	29,6	1,03
HIV support	To what extent does access to treatment promote HIV disclosure?	10,7	1,24
PMTCT	Identification of best strategies for provision and monitoring of CD4 testing and treatment for pregnant and breastfeeding women	10,7	1,18
PMTCT	Factors influencing the retention of care in the first 12 months of life	14,3	1,29
PMTCT	To what extent are recommendations regarding feeding options are understood by the service providers at different levels in the health delivery system	32,1	1,29

During the first day of the workshop presentations were provided by the key note speaker focusing on knowledge gaps in the following relevant research areas: Prevention of mother-to-child transmission of HIV (PMTCT) and early infant diagnosis of HIV (EID); HIV treatment, care and support; HIV monitoring and evaluation; Epidemiology and surveillance; HIV and tuberculosis (TB).

In the following research questions for the above mentioned areas identified by the participants of the workshop are presented:

### Inventory of HIV/AIDS-relating research questions

**Prevention of mother-to-child transmission of HIV and early infant diagnosis of HIV**: Nigeria belongs to the 10 highest burden countries with 210,000 HIV positive pregnant women in 2008 [4]. PMTCT is a focused intervention that should begin ideally prior to pregnancy and ends at delivery or slightly thereafter. Eliminating paediatric HIV/AIDS is achievable and PMTCT is considered an essential maternal, newborn and child health care. Four main comprehensive approaches to PMTCT include; primary HIV prevention among women of reproductive age, prevention of unintended pregnancies among HIV positive women, prevention of HIV transmission from infected pregnant women to their children, and treatment, care and support for HIV infected women, their children, and families [4]. The main research questions identified are summarized in Table 2.

**Table 2:** PMTCT/EID-related research questions

Area	Research questions
**PMTCT uptakeand retention**	What strategies can influence acceptance and uptake of PMTCT services?
	What community strategies can effectively increase PMTCT uptake?
	What are the strategies to ensure retention of pregnant women throughout the PMTCT cascade especially delivery in health facilities?
	What are the roles of community women (e.g. market women) in PMTCT?
	What are the socio-cultural (religious) factors that hinder access to PMTCT services?
	What is the role of male involvement in PMTCT?
	What is the availability of uniform PMTCT protocols?
	What are the challenge of integration of PMTCT services and conventional health services?
**Family planning**	What are the factors that influence the decision on HIV positive pregnant women to get pregnant?
	What are the contraceptive needs and choices of HIV positive women of reproductive age group?
**Pregnancy/delivery**	Why do women prefer to deliver with TBAs?
	What can be the roles of the traditional birth attendants in PMTCT?
	What is the role of the private sector in PMTCT?
**Feedingoptions**	How best can HIV positive pregnant women be supported to adhere with their chosen infant feeding methods?
**EID**	How to ensure provision of early detection and confirmatory test facilities at all levels of care?
	What is the level HIV drug resistance in HIV-positive children in Nigeria?

**HIV Treatment Care and Support**: Of the estimated 1.5 million PLHIV eligible for treatment 29.8% at the end of 202 were on treatment for both adults and children. Adherence challenges among patients attending ART clinics in Nigeria [1]. There are great disparities in access to health care among different population groups in Nigeria, with those in rural areas having limited access to health care due to low level of education, socio-cultural barriers and poor infrastructure. It is estimated that only about a quarter (26%) of women in rural areas deliver with the assistance of a doctor or nurse/midwife, in contrast to about 59% of women in urban areas. Using number of births attended to by skilled health workers, as a proxy, it is estimated that only 34% of the general population have access to basic health care. Significantly, unlike most of sub-Saharan Africa, rural areas in Nigeria have a higher HIV prevalence than urban areas, which compounds the issue of accessibility to health care services given the high rural – urban disparities in the country [5].

Nigeria has established the following targets with regard to HIV care and treatment [5]: 1) Increase access to HIV counselling and testing (HCT) 80% by 2015; 2)Place 1.5 million people living with HIV/AIDS (PLWAs) on antiretroviral treatment (ART) by 2015 Decrease the prevalence and incidence of sexually transmitted infections (STIs) among PLWAs to 50% by 2015; 3) Place at least 400,000 new patients on ART in 2013.

A wide range of research questions have been identified which are presented in Table 3.

**Table 3:** Identified research areas for HIV care and support

Area	Research topics
**Diagnosis**	What clinical and/ or laboratory approaches can be employed to optimize diagnoses of HIV/AIDS?
	What is the best time and method of disclosure to paediatric and adolescent patients (sero-discordant couples, polygamous setups)?
	What are the determinants of the poor linkage from testing to enrolment in care and treatment?
	How do we improve HCT uptake to meet the target of presidential order of putting 400,000 new PLWAs on ART?
**Antiretroviral treatment uptake and monitoring**	What are the challenges and barriers to treatment seeking among different populations of PLWA?
	What is/are the best model(s) for delivering ART services, e.g. de-centralization & integration of ART services with other services?
	What are the determinants for default of ART? What is the best treatment model for optimal adherence; Facility based or community based?
	How can resources for monitoring ART be optimized,e.g. use of targeted viral load?
	What treatment regimens are most potent, cost effective and improve adherence?
	What is the contribution of private health facilities to ART services in Nigeria; what are the quality and spectrum of services rendered?
	ARV drug resistance, what are the early warning signs from facilities?
	Efavirenz and teratogenicity; are there cases associated with teratogenicity in past?
	What is the prevalence of HIV-related malignancies; what is their impact on presentation?
	What is the prevalence of co-infection in certain groups /sub-populations, e.g. prisoners, (para) military, and high at risk groups?
**Co-morbidity**	How can the diagnosis of opportunistic infections be optimized? What clinical or lab approaches (biomarkers, clinical algorithm)?
	What is the impact of high attrition of trained health care workers on ART services? Doesitaffect Quality of care?
	What protection and support strategies are available for health care workers; what is the outcome of post exposure prophylaxis (PEP)?
**Service**	What are the prospects and challenges of integration of HIV/AIDs treatment into mainstream clinical services?
	Do healthcare workers use the national guidelines for HIV/AIDs treatment?
	What system of quality improvement for care is in place? Is it working well? Has it improve quality of care? Whatarethechallenges?
	Community home-based care: what is the quality? What are the sources and channel of resources?
	What are the models of ART services decentralization in Nigeria and their effectiveness?
	How to enhancing the success rate of community-based referral system in linking HIV positive people with treatment centres?
	Expanding the provision of ART And PMTCT services through engagement of private health facilities in the national HIV response in Nigeria
**Drugs andconsumablesupply**	What is impact on drugs delivery and consumables supply? What are the challenges? How have tjese factors affected quality of care?
	What is the level HIV drug resistance in HIV-positive children in Nigeria?

### Epidemiology and surveillance

At the end of 2012, about 3.5 million people were living with HIV in Nigeria. This accounted for 9.4% of the global burden and 13.6% of the burden in Sub-Saharan Africa. The median national prevalence decreased in the recent years, however, the absolute number of PLWHIV increased by half a million and AIDS mortality has increased to about 217,000 annual death attributed to AIDS accounting for 13.7% of global AIDS deaths and 19.4% of AIDS deaths in Sub-Saharan Africa. Actually the following surveillance efforts in HIV are on-going: 1) HIV incidence study; 2) Surveillance of HIV drug resistance; 3) Early Warning Indicator Survey (EWI) for HIV drug resistance; 4) Determination of level of HIV infections among TB clients.

Identified knowledge gaps with regard to surveillance and epidemiology are listed in Table 4.

**Table 4:** Epidemiology and Surveillance gaps for HIV/AIDS

Research area	Topic
**Data availability and dissemination**	What data dissemination plans are in place?
**Surveillance**	What are the determinants of limited activities in STI surveillance?
	What are the challenges of the AIDS case reporting system?
	What is the HIV prevalence among health care providers at hospitals?
	What are the factors contributing to the different HIV prevalence on the state level?
	How can a HIV drug resistance surveillance system be established?
**Epidemiology**	What are the factors associated with high and low HIV prevalence rates at ANC sentinel sites?
	What is the association between HCT services uptake and the epidemiology of HIV/AIDS among women attending ANC in high and low sentinel sites in Nigeria?
	What factors are associated with high or low epidemics in four highest and lowest sentinel sites in Nigeria?

**HIV and tuberculosis**: The spread of HIV has fuelled the tuberculosis epidemic. Tuberculosis is a major cause of death among people living with HIV and accounts for about 22% of HIV-related deaths globally. In 2012, 1.1 million of the 8.8 million incident cases of TB worldwide were among people living with HIV [6]. HIV is one of the main reasons for failure to meet tuberculosis control targets in high HIV settings. Identified research question raised regarding TB-HIV co-infection are summarized in Table 5.

**Table 5:** Research questions identified for HIV-TB-co-infection

Area	Research topics
**Diagnosis**	How to improve diagnosis of tuberculosis among HIV infected individual?
	What is extent of Isoniazid preventive therapy (IPT) coverage? What are the challenges of instituting IPT?
	What is the burden of and diagnostic challenges of non-tuberculous mycobacterium among HIV-infected individuals?
	What are the best strategies for co-management and referral for TB and HIV cases after diagnosis?
	What are the TB type and molecular characterics among HIV positive TB patients?
**Co-management**	What is the extent of TB-HIV service integration? What models are being used and what are the challenges to optimal integration of these services?
	What is the level of TB infection control in our ART facilities? What are the challenges in TB infection control?
	Are there infection control committees & guidelines in place?
**Treatment**	How do we improve adherence to medications in patients with TB/HIV/Hepatitis B? Can provision of DOTS (at facility and community levels) improve outcome of treatment? Does provision of treatment to partner improve outcome?
	How do we reduce attrition rates in our ART programs? What factors contribute to this? How do we tract these patients? How do we optimize retention of these patients within the programs?
	What is the treatment success rate of TB-treatment in PLWAs?
	What are socio-economic and socio-cultural factors hindering co-treatment of TB and HIV?
	What are the determinants of the high rate of loss to follow-up? How do we tract the patient (phone number)?
	What are the challenges to the provision of ART/PMTCT services in private health facilities?
**Drug resistance**	What is the burden of HIV-drug resistance and Drug resistant TB(DR-TB)?
	What are the challenges in TB-HIV co-infection management in the presence drug resistance?
**Co-infection**	What is the outcome of managing TB-HIV co-infection in the presence of hepatitis? Whatarethechallengesoftreatment?
**Support**	What is the role played by patients support groups in improving socio-economic status of PLWAs?

**Scoring of research questions**: Scoring exercise was done for a limited number of research questions for the following areas: Tuberculosis and HIV-co-infection, PMTCT and HIV care and support. For each research question an average score was calculated from the scores of the 15 questions (Table 6).

**Table 6:** Scoring of HIV/AIDS-related research questions

Research area	Research topic	Average scoring
**PMTCT**	Investigation of socio-cultural and religious factors hindering access to PMTCT services	6.6
	Assessment of the provision of early detection and confirmatory test in health facilities	6.4
	Identification of best strategies for the provision and monitoring of CD4 test and treatment during pregnancy and breastfeeding	6.2
	Determination of uptake of PMTCT	6.1
	Factors that determine acceptance of exclusive breastfeeding among HIV-positive mothers	5.5
**TB/HIV**	Incidenceof MDR-TB	8.4
	Assessment of TB treatment success in HIV-positive patients	7.4
	Socio-economic and socio-cultural factors hindering the co-management of TB and HIV	6.7
**Care andtreatment**	Assessment of challenges of optimizing quality in care	8.0
	Factors associated with adherence to antiretroviral drugs	7.9
	What are the contributions of the private health sector to ART services	7.9
	What are the challenges and prospects of optimizing gene expert in TB diagnosis among TB suspect HIV positive patients?	7.8
	Pharmacovigilance: What is the level of integration in ART services in Nigeria?	7.8
	What are the factors associated with compliance to National Guidelines?	7.7
	What are the outcomes and challenges of post-exposure prophylaxis among health care workers?	7.6
	What are the challenges of IPT utilization in ART facilities?	7.6

**Development of research proposals**: Eighteen residents submitted concept notes on HIV/AIDS-related research ideas and developed draft study proposals which were presented and discussed with the workshop participants.

## Discussion

The workshop on HIV/AIDS-related research questions was the first workshop held by the Nigeria Field Epidemiology and Training Programme to establish an inventory of research questions which can be addressed by the residents within their training periods. This inventory will help to increase HIV/AIDS-related activities of NFELTP which are in accordance with research needs in Nigeria and PEPFAR goals.

Overall the participants agreed that the workshop was satisfactory (4.4; maximum score 5), that the work objectives were met (4.5) and that the workshop was useful for the job (4.6). However, time constraints were criticized by the participants. In the comments participants observed three days was too short, that more time should be given to the group work and the presentations of the group work. Some participants regarded the material provided for the preparation of the workshop as not useful. The interactive approach used in this workshop allowing an exchange between the residents of different cohorts and the facilitators was rated positively.

The workshop on HIV/AIDS-related research questions was the first workshop held by the Nigeria Field Epidemiology and Training Programme to establish an inventory of research questions which can be addressed by the residents. This inventory will help to increase HIV/AIDS-related activities of the programme which are in accordance with research needs in Nigeria.

## Conclusion

The next step should be the scoring and prioritization of identified research questions. A two to three days workshop needs to be organized. Possible participants for the workshop should be representatives from National HIV/AIDS and Sexually Transmitted Diseases Control Programme (NASCP), National Agency for the Control of AIDS (NACA), academic supervisors from Ahmadu Bello University, Zaria and University of Ibadan, the program director and staff from Nigeria Field Epidemiology and Training Programme (NFELTP), Centers for Disease and Prevention Nigeria (CDC) staff and residents working in the area of HIV/AIDS. Further action points are: develop full protocols for some of the research areas and carry out the research; support a national HIV research prioritization workshop and develop research priority areas for other public health issues.
